# Comparative in vivo characterization of newly discovered myotropic adeno-associated vectors

**DOI:** 10.1186/s13395-024-00341-7

**Published:** 2024-05-03

**Authors:** Jacqueline Ji, Elise Lefebvre, Jocelyn Laporte

**Affiliations:** Institute of Genetics and Molecular and Cellular Biology (IGBMC), INSERM U1258, CNRS UMR7104, University of Strasbourg, IGBMC, 1 rue Laurent Fries, Illkirch, 67404 France

**Keywords:** Adeno-associated virus, AAV, Tissue targeting, Myotropism, Gene therapy, Muscle, Myopathy, Heart, Liver

## Abstract

**Background:**

Adeno-associated virus (AAV)-based gene therapy is a promising strategy to treat muscle diseases. However, this strategy is currently confronted with challenges, including a lack of transduction efficiency across the entire muscular system and toxicity resulting from off-target tissue effects. Recently, novel myotropic AAVs named MyoAAVs and AAVMYOs have been discovered using a directed evolution approach, all separately demonstrating enhanced muscle transduction efficiency and liver de-targeting effects. However, these newly discovered AAV variants have not yet been compared.

**Methods:**

In this study, we performed a comparative analysis of these various AAV9-derived vectors under the same experimental conditions following different injection time points in two distinct mouse strains.

**Results:**

We highlight differences in transduction efficiency between AAV9, AAVMYO, MyoAAV2A and MyoAAV4A that depend on age at injection, doses and mouse genetic background. In addition, specific AAV serotypes appeared more potent to transduce skeletal muscles including diaphragm and/or to de-target heart or liver.

**Conclusions:**

Our study provides guidance for researchers aiming to establish proof-of-concept approaches for preventive or curative perspectives in mouse models, to ultimately lead to future clinical trials for muscle disorders.

**Supplementary Information:**

The online version contains supplementary material available at 10.1186/s13395-024-00341-7.

## Background

Recombinant adeno-associated virus (AAV)-based vectors represent a main tool to establish gene therapy for efficient transduction of large anatomically distributed tissues, as needed for muscle diseases. However, clinical development faces poor transduction efficiency and off-target toxicity. Here, we compared the muscle transduction efficiency and the liver de-targeting capacity of the latest myotropic AAV serotypes to better allow their selection according to the desired pre-clinical applications.

These AAV vectors consist of a single stranded DNA molecule protected by a protein capsid and present many advantages for clinical use in human, including non-pathogenicity, a wide tropism, genomic stability and long-term transduction, offering a promising tool for treating a plethora of diseases [1–5]. Some diseases, as muscle diseases, represent a particular challenge as a large mass of tissue within the body should be transduced. Indeed, skeletal muscle accounts for nearly half of lean body mass in mammals. Recently, ELEVIDYS, a first AAV-based gene therapy product treating Duchenne muscular dystrophy (DMD) has been approved by FDA [6]. However, several obstacles remain for the development of such therapeutic strategy, such as (1) the huge amount of vector needed to transduce the entire muscular system [7], (2) the toxicity caused in other organs and especially in the liver and (3) the pre-existence of immunity against AAVs [8]. Indeed, while high dose (3E + 14vg/kg) of AAV8-MTM1 (AT132) in patients with the severe myotubular myopathy correlated with muscle amelioration, 4 patients died from hepatic complications including cholestasis following the treatment, leading to the halting of the clinical trial (NCT03199469) [9, 10]. For Duchenne muscular dystrophy, a single dose of AAV9-micro dystrophin (5E + 13vg/kg) was tested in non-ambulatory adolescent DMD patients and transient renal dysfunction and hematological complications appeared few days post-administration [11]. In the case of spinal muscular atrophy, a research study revealed that among 100 patients, 34 individuals exhibited hepatotoxicity as an adverse effect subsequent to the administration of AAV9-SMN1 (ZOLGENSMA) vector dose at 1.1E + 14 vg/kg [12]. Noteworthy, the AAV doses used in clinics are high and a challenge to produce for human application.

Thus, recent pre-clinical developments tried to tackle the transduction efficiency and tissue specificity by generating and testing novel AAV serotypes. Several serotypes - AAV6 [13], AAV8 [14], AAV9 [15], AAVrh74 [16], AAVpo1 [17] - have a natural tropism for muscles, and most were previously tested in clinical trials. Recently, novel AAV serotypes were developed to increase their muscle-specific transduction efficiency. Two main approaches were followed: rational design modification of the capsid through insertion of a peptide sequence presenting high affinity for a known surface receptor [18–21], and directed evolution through the introduction of random capsid mutations and several rounds of in vivo selection [22–26]. Using the latter strategy, Tabebordbar et al. [27] and Weinmann et al. [28] generated myotropic AAVs, called MyoAAV and AAVMYO, respectively. These two myotropic AAVs share the “RGD” motif -Arginine, Glycine, Aspartic Acid- which is known to interact with the integrin family [29–33], some of its members being expressed on the surface of muscle cells [34–36]. In addition to their tropism for skeletal and cardiac muscles, MyoAAV and AAVMYO showed a liver de-targeting effect compared to AAV9, making these two AAVs promising tools for gene therapy in muscle diseases.

**Fig. 1:**
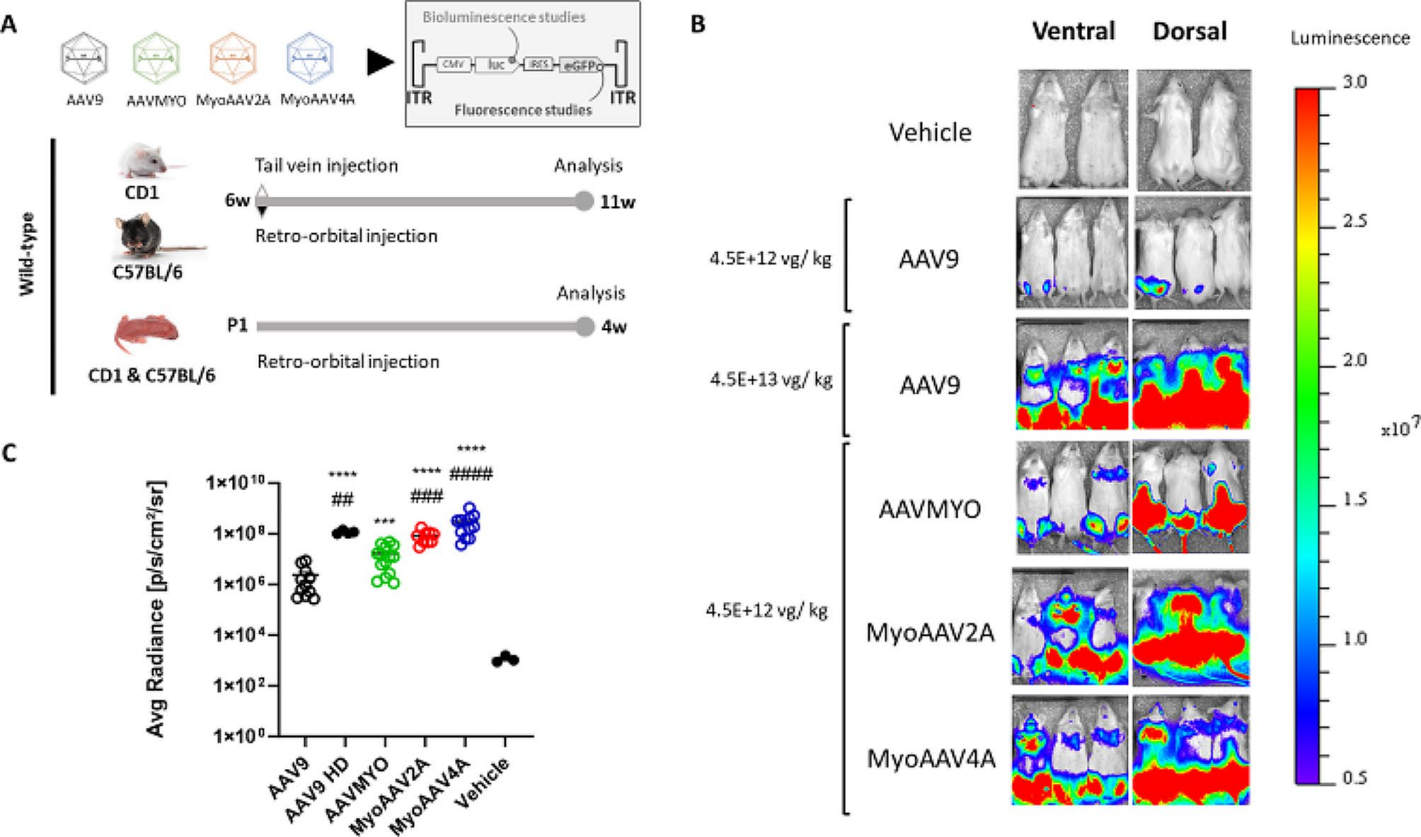
Study design and comparison of in vivo luminescence levels between AAV9 and myotropic AAVs in adult mice 5w after systemic injection. (**A**) Study design. Genes coding for luciferase and eGFP are contained in the cytomegalovirus (CMV)-driven transgene separated by an IRES (Internal Ribosome Entry Site) sequence that allows translation of both proteins. Wild-type (WT) CD1 and C57BL/6 mice have been injected at 6w (weeks) of age and analysis performed 5w after injection, or at post-natal day 1 and analyzed 4 weeks later. (**B**) Expression of whole-body in vivo luminescence in WT CD1 mice systemically injected with 4.5E + 12 vg/kg (viral genome / kilogram) (low dose) of AAV9-, AAVMYO-, MyoAAV2A-, MyoAAV4A-CMV-luc-IRES-eGFP or 4.5E + 13 vg/kg (high dose, HD) of AAV9-CMV-luc-IRES-eGFP. Color scale: 5E + 6–3E + 7 photons per second per centimeter square per steradian (p/sec/cm²/sr). (**C**) Quantification of in vivo luminescence in CD1 mice injected with AAV9-, AAVMYO-, MyoAAV2A-f, MyoAAV4A- at 4.5E + 12 vg/kg or AAV9-CMV-luc-IRES-eGFP at 4.5E + 13 vg/kg (HD) taken at 11 weeks. Luminescence is quantified by measuring the average radiance (p/sec/cm²/sr). Data are presented as mean values +/- SEM (*n* = 4–13). One-way ANOVA with Tukey correction. ***p < 0.001 and ****p < 0.0001 versus AAV9; ##*p* < 0.01, ###*p* < 0.001, ####*p* < 0.0001 versus AAVMYO

Overall, main bottlenecks for the translation of AAV gene therapy into clinic for muscle diseases are the poor transduction efficiency, the need to target a large tissue mass, and toxicity due to off-target transduction. In the present study, a comparative analysis was conducted to assess latest myotropic AAVs in wild-type mice. While the transduction efficiency of these myotropic AAVs has been individually reported in the literature, a comparative analysis has not been performed. We compared AAV9, MyoAAV2A and 4 A, and AAVMYO transduction efficiency in different muscles and organs, at two distinct systemic injection time points (postnatal day 1 and 6 weeks) and in two different mouse strains (Fig. 1A).

## Materials and methods

### Animals

Experiments were performed using male and female C57BL/6 or CD1 background mice at 6 weeks (6w) or at post-natal day 1 (1 d.p.n.) available at the Institut Clinique de la Souris (ICS). C57BL/6 adult mice received a retro-orbital injection of 4.5E + 12 vg/kg (viral genomes per kilogram; low dose) or 4.5E + 13 vg/kg (high dose). CD1 adult mice were intravenously injected into the tail vein with either 4.5E + 12 vg/kg or 4.5E + 13 vg/kg. Additionally, 1 d.p.n. mice received a retro-orbital injection with a total dose of 4.1E + 12 vg/kg. All animals were bred and accommodated within a controlled environment of specified pathogen-free conditions, under 12-hour light/dark cycles, within a room regulated for temperature and humidity. Animal care and experimentation were in accordance with French and European guidelines and approved by the institutional ethics committee Com’eth (project number APAFIS #39608-2022102815593282) and accredited by the French Ministry for Superior Education and Research and in accordance with the Directive of the European Parliament (2010/63/EU). Mice were euthanized in accordance with national and European legislations on animal experimentation.

### AAV construction and production

Recombinant AAV9, AAVMYO, MyoAAV4A and 2 A were generated by the molecular biology and virus facility at IGBMC, by a triple transfection of HEK293T/17 cell line with the expression plasmid pAAV-CMV-luc-IRES-GFP-SV40pA (Addgene #105,533, Massachusetts, US) and the auxiliary plasmids pHelper (Agilent, California, US) and the capsid plasmids pAAV2/9 P0008 (Penn Vector Core, Pennsylvania, US), pAAVMYO, pMyoAAV2A or pMyoAAV4A (all created by modifying the AAV2/9 capsid by insertion of peptide sequences made available in the literature [27, 28]). Recombinant adeno-associated viruses (rAAV) were harvested 48 h after transfection from cell lysates treated with 100U/mL Benzonase (Merck, New jersey, US). All AAV serotypes were purified by Iodixanol gradient ultracentrifugation (OptiprepTM, Axis Shield) followed by dialysis and concentration against Dulbecco’s PBS containing 0.5mM MgCl2 using centrifugal filters (Amicon Ultra-15 Centrifugal Filter Device 100 K). Viral titers were determined either by qPCR using the LightCycler480 SYBR Green I Master (Roche, Switzerland) and primers targeting eGFP sequence or by digital droplet PCR (ddPCR) [37]. Viruses were stored at -80 °C until use.

### In vivo luminescence imaging

For in vivo imaging, mice injected at 6 weeks of age received a subcutaneous administration of D-luciferin in PBS at a dose of 150 mg/kg of body weight. Subsequently, they underwent bioluminescence imaging analysis using an optical imaging system (IVIS Lumina XRMS). All images containing raw data were processed using the Living Image software (Perkin Elmer, Massachusetts, US). The freehand tool was employed to extract the total luciferase signals from the whole body of the 6-week injected mice. Data were exported in photons per second per square centimeter per steradian (p/sec/cm²/sr) and represented as a pseudo-color overlay onto a grayscale image of the animal.

### DNA/ RNA extraction and cDNA synthesis

Frozen tissues samples were mechanically lysed with a Precellys homogenizer (Bertin technologies, France) in TRIzol reagent (ThermoFischer #15,596,026, Massachusetts, US) and processed according to the manufacturer’s instructions to obtain total RNA. DNA were extracted using the Puregene Tissue Kit (Qiagen #158,063, Germany). Both total DNA and RNA concentration were determined by spectrophotometry (Nanodrop 2000, ThermoScientific, Massachusetts, US). cDNA was produced from RNA using SuperScript IV Reverse Transcriptase (ThermoFischer #18,090,010, Massachusetts, US).

### Determination of vector copy numbers and mRNA expression by quantitative real-time PCR

For vector copy number quantification per diploid genome, 12 µL qPCR reaction was performed containing DNA extraction, SYBR Green Master Mix I (Roche #04707516001, Switzerland), *eGFP*-containing vector genomes primers (5’-GAC GAC GGC AAC TAC AAG A-3’ (forward); 5’-CAT GAT ATA GAC GTT GTG GCT-3’ (reverse)) and *Rps11* housekeeping gene primer (5’-CGC GTG GTG AAT AAG GAA GC-3’ (forward), 5’-GTA AGC ACG CTC CGT CTG AA-3’ (reverse)). Standard curves were established for determination of vector copy number by using transgene plasmid and eGFP primers. The same procedure was applied for the copy number of the housekeeping gene. VCN of each sample was normalized to the copy number of the housekeeping gene. For quantification of eGFP mRNA expression, cDNA from retro-transcription of RNA extraction was used to perform qRT-PCR. Results for all AAVs were presented as fold change mRNA expression compared to AAV9.

### Luciferase activity assay

After 5 weeks post AAV injections, animals were euthanized and organs (TA, diaphragm, heart, liver) were harvested. The collected organs were rapidly frozen in liquid nitrogen and subsequently stored at -80 °C. Once frozen, the organs were pulverized into a powder and then 250 µL of lysis buffer (Promega #E4030, Wisconsin, US) was added. The samples were vortexed at room temperature for 15 min, then underwent three cycles of freezing in liquid nitrogen and thawing using a 37 °C water bath. Following this, samples were centrifuged for 3 minutes at 10,000 x g, and the resulting supernatant was retained for subsequent analysis. Luciferase activity was measured using a luminometer (Centro XS^3^ LB 960). Specifically, 100 µL of luciferase substrate (Promega # E4030, Wisconsin, US) was added to 20 µL of extracted tissues, and the relative light units (RLU) were measured 5 s after mixing. The quantification of luciferase activity was determined by normalizing RLU to protein concentration in µg.

### Immunofluorescence

C57BL/6 mice were injected retro-orbitally with 4.5E + 13vg/kg of AAV9-, AAVMYO-, MyoAAV2A-, MyoAAV4A-CMV-luc-IRES-eGFP and tibialis anterior muscles of each injected mouse were collected and 8 μm thick muscle cross-sections were prepared for immunofluorescence studies. After being fixed with 4% paraformaldehyde and permeabilized with 0.02% Triton 1X, transversal TA sections were washed with PBS 1X and blocked 1 h with BSA 3% (bovine serum albumin) in PBS to avoid nonspecific binding. After, BF-F3 and SC-71 (Developmental Studies Hybridoma Bank, Lowa, US) diluted at 1:50 and eGFP (Invitrogen #A-11,122, US) diluted at 1:100 were used as primary antibodies to detect muscle fiber type IIb and transgene vector expression, respectively. Sections were incubated with primary antibodies overnight at 4 °C in a humidified atmosphere. After series of washing with PBS 1X, sections were treated with secondary antibodies IgM DyLight 405 goat anti-mouse (dilution 1:100), IgG1 Cy5 goat anti-mouse (dilution 1:100) or Alexa Fluor 488 donkey anti-rabbit (dilution 1:400) and during 1 h at room temperature. After series of washing, sections were mounted with ProLongTM Gold antifade reagent (Invitrogen #P36934, Massachusetts, US) and air-dried before imaging using a microscope (Zeiss, Germany).

### Statistical analysis

All data were expressed as mean +/- SEM. Statistical analysis was performed using the GraphPad Prism program 9.5.1 (GraphPad Software, San Diego, CA). Data were analyzed by an unpaired one-way or two-way ANOVA with respectively Tukey or Bonferroni correction after verification of normal or lognormal distribution using D’Agostino & Pearson test or Shapiro-Wilk test. P values < 0.05 were considered significant.

## Results

### AAVMYO, MyoAAV2A, and MyoAAV4A show higher whole-body transduction efficiency compared to AAV9

To assess the transduction efficiency and organ distribution of myotropic AAVs (AAVMYO, MyoAAV2A, MyoAAV4A) in comparison to AAV9, a cytomegalovirus (CMV)-driven transgene containing genes coding for luciferase and eGFP was inserted in these AAV serotypes for in vivo and ex vivo analyses (Fig. 1A). Bioluminescence was first performed in vivo to measure luminescence intensity based on luciferase activity in 6-week-old CD1 mice injected via tail vein with a low dose of 4.5E + 12 vg/kg (equivalent of 1E + 11 vg per mouse) and measured 5 weeks post injection. The doses used were calculated from qPCR titration, and the results obtained by ddPCR are shown in Figure S1.

Most injected animals showed bioluminescence in the upper and lower limb regions in the prone position and hindlimb region in the back position, suggesting several muscle groups were transduced (Fig. 1B). In addition, luminescence was visible in the plexus region in MyoAAVs-injected mice, potentially the heart. Also, luminescence was visible in the upper abdominal region in only AAV9-injected mice after 1 week, suggesting a potential expression in liver, while the signal disappeared after 5 weeks (Figure S2). The intensity of luminescence, measured by determining the average whole-body radiance, was higher in AAVMYO, MyoAAV2A, and MyoAAV4A compared to AAV9 (Fig. 1C). Luminescence with MyoAAV2A- and MyoAAV4A was respectively 5 and 18 times higher than with AAVMYO. No differences of intensity of bioluminescence were observed between male and female mice, as well as between retro-orbital and tail-vein injections (Figure S3-S4).

Later, to compare muscle transduction efficiency between all the AAVs, we next injected a higher dose (4.5E + 13 vg/kg) of AAV9 into a separate cohort of mice. This high dose of AAV9 showed a similar pattern of bioluminescence localization as in AAVMYO, MyoAAV2A, and MyoAAV4A-injected mice at the 10 times lower dose (Fig. 1B). Although no significant difference in average whole-body radiance was observed between AAV9 HD, MyoAAV2A, and MyoAAV4A, the results showed 7-times lower luminescence with AAVMYO (1.7E + 7 ± 4.2E + 6 p/sec/cm²/sr) compared to AAV9 HD (1.2E + 8 ± 9.6E + 6 p/sec/cm²/sr).

In general, a stronger luminescence related to transgene expression was observed in AAVMYO, MyoAAV2A, and MyoAAV4A compared to AAV9 at the same dose, mainly located in several muscle regions.

### MyoAAV2A and MyoAAV4A present higher transduction in leg muscle and heart while AAVMYO is most efficient in diaphragm

**Fig. 2:**
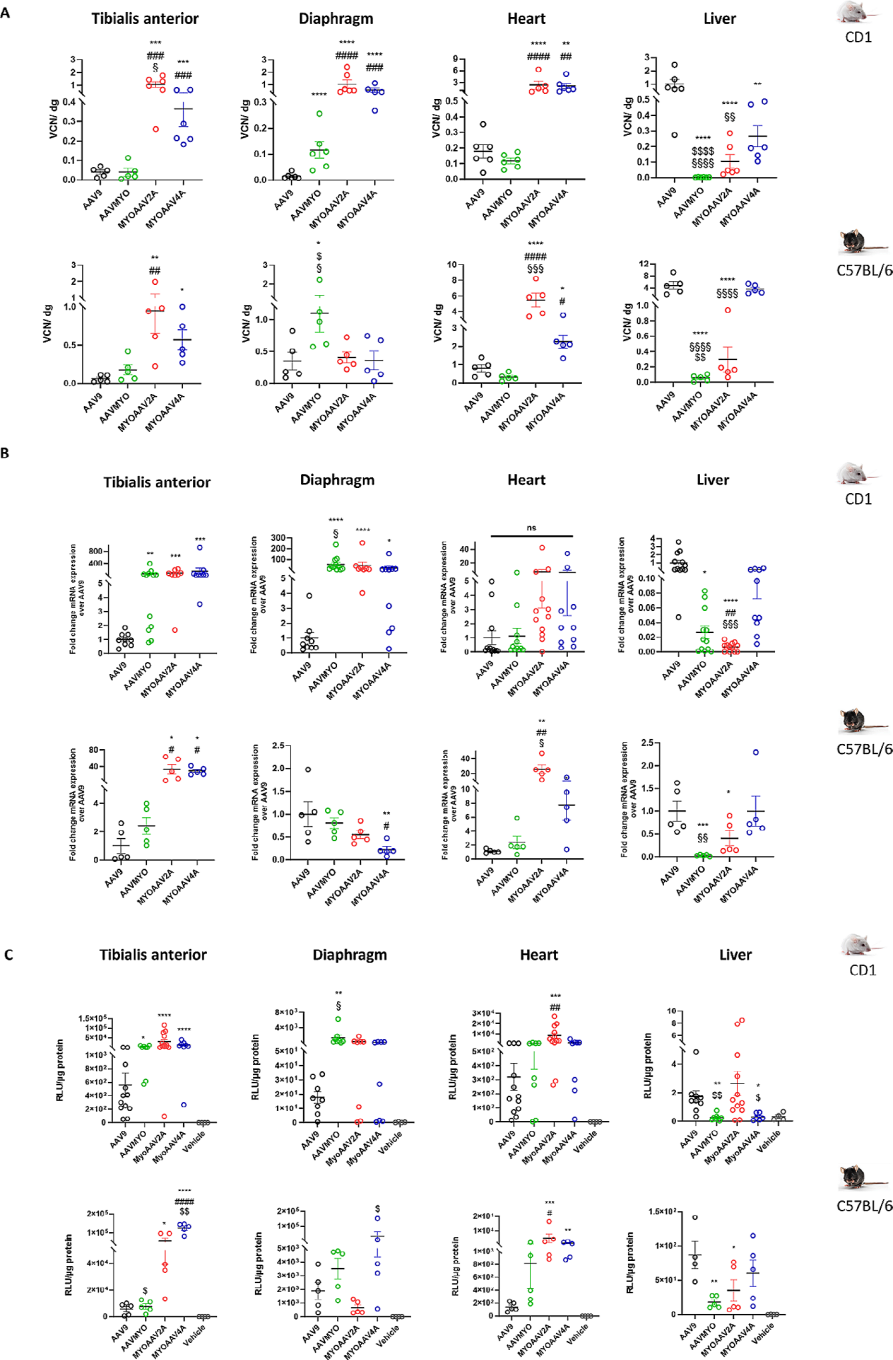
Comparison of transduction efficiency between AAV9 and myotropic AAVs in several organs from wild-type adult mice. Mice were systemically injected at 6-week-old and organs collected 5 weeks later. (**A**) Quantification of vector copy number per diploid genome of AAVMYO-, MyoAAV2A-, MyoAAV4A- and AAV9-CMV-luc-IRES-eGFP in WT CD1 and C57BL/6 mice in tibialis anterior leg muscle, diaphragm, heart and liver. Data are presented as mean values +/- SEM (*n* = 5–6). One-way ANOVA with Tukey correction. *p < 0.05, **p < 0.01, ***p < 0.001, ****p < 0.0001 versus AAV9 ; #*p* < 0.05, ##*p* < 0.01, ###*p* < 0.001, ####*p* < 0.0001 versus AAVMYO ; $*p* < 0.05, $$$$<0.0001 versus MyoAAV2A ; §*p* < 0.05, §§*p* < 0.01, §§§§*p* < 0.05 versus MyoAAV4A. (**B**) Quantification of eGFP mRNA fold change expression of AAVMYO-, MyoAAV2A-, MyoAAV4A- and AAV9-CMV-luc-IRES-eGFP in WT CD1 and C57BL/6 mice in different organs. Data are presented as mean values +/- SEM (*n* = 5 for C57BL/6 mice, *n* = 9–12 for CD1 mice). One-way ANOVA with Tukey correction. *p < 0.05, **p < 0.01, ***p < 0.001, ****p < 0.0001 versus AAV9; #*p* < 0.05, ##*p* < 0.01, ####*p* < 0.0001 versus AAVMYO; §*p* < 0.05, §§*p* < 0.01 §§§§*p* < 0.0001 versus MyoAAV4A. (**C**) Quantification of luciferase activity of AAVMYO-, MyoAAV2A-, MyoAAV4A- and AAV9-CMV-luc-IRES-eGFP in WT CD1 and C57BL/6 mice in different organs. Data are presented as mean values +/- SEM (*n* = 5 for C57BL/6 mice, *n* = 9–13 for CD1 mice). One-way ANOVA with Tukey correction. *p < 0.05, **p < 0.01, ***p < 0.001, ****p < 0.0001 versus AAV9; ##*p* < 0.01, ####*p* < 0.0001 versus AAVMYO ; $*p* < 0.05, $$*p* < 0.01 versus MyoAAV2A ; §*p* < 0.05 versus MyoAAV4A

A pilot study was performed with different AAV serotypes in CD1 mice and VCN/dg was measured. MyoAAV2A and MyoAAV4A showed a higher vector copy number (VCN) in TA and heart compared to AAVMYO in both strains, while no increase was observed for AAVMYO compared to AAV9 in these tissues (Fig. 2A). In diaphragm, the three myotropic AAVs exhibited a strong increase in VCN compared to AAV9.

Next, we used other mouse cohorts to compare two different routes of systemic injection and also two mouse strains with different genetic backgrounds. Several tissues (tibialis anterior-TA, diaphragm, heart, and liver) were collected 5 weeks after systemic AAV injection with a low dose (4.5E + 12 vg/kg) in 6-week-old CD1 (tail vein) and C57BL/6 (retro-orbital) mice and eGFP mRNA level and luciferase activity were measured.

The level of eGFP mRNA expression in TA muscle was significantly increased for all myotropic AAVs compared to AAV9 in CD1 mice while it was the case only for MyoAAV2A and MyoAAV4A in C57BL/6 mice with 32 and 28 times respectively (Fig. 2B). In diaphragm, AAVMYO and MyoAAV2A correlated with increased mRNA expression in CD1 mice, while MyoAAV4A showed a decrease mRNA level in C57BL/6 mice compared to AAV9. In heart, only MyoAAV2A showed a mRNA increase in C57BL/6 mice (6.5 times) (Fig. 2B). In C57BL/6 mice injected at high dose, AAVMYO demonstrated a significant increase in transgene mRNA level in TA but not in heart compared to AAV9 (Figure S5).

In correlation with the eGFP mRNA expression, quantification of luciferase activity representing the transgene expression showed a higher transduction efficiency for MyoAAV2A- and MyoAAV4A- compared to AAV9- and AAVMYO-injected CD1 and C57BL/6 mice in TA muscle (Fig. 2C). MyoAAV2A presented the highest luciferase activity in TA in CD1 (3.2E + 4 ± 1.1E + 4 RLU/µg total proteins) mice, whereas MyoAAV4A appeared more efficient in C57BL/6 mice (1.3E + 5 ± 1.2E + 4 RLU/µg total proteins). For diaphragm, AAVMYO was the most efficient to transduce CD1 mice. In heart, MyoAAV2A achieved the highest luciferase activity in both mouse strains. Variations were observed between strains, with a global loss of transduction efficiency in all tissues in CD1 compared to C57BL/6 mice. Overall, the highest expression was noted for AAVMYO in diaphragm (79-fold compared to AAV9).

In conclusion, MyoAAV2A and MyoAAV4A showed the most efficient transduction in TA muscle and heart, whereas AAVMYO was the most efficient in diaphragm among all tested AAVs.

### AAVMYO, MyoAAV2A and MyoAAV4A transduce more efficiently type IIb myofibers compared to AAV9

To investigate the basis for the better muscle transduction with myotropic AAVs, we investigated if myotropic AAVs show different transduction patterns depending on myofiber types. In mouse muscles, there are barely slow (oxidative) type I fibers, while type IIa are more oxidative than type IIb [38]. Indeed, it has been demonstrated that AAV9 shows a preference for type IIa and IIX myofibers, while it poorly transduces type IIb myofibers [39]. Immunofluorescence was performed on cross-sections of TA muscles systemically transduced with the different AAVs at 4.5E + 13 vg/kg to identify eGFP signal and type IIa and IIb myofibers.

**Fig. 3:**
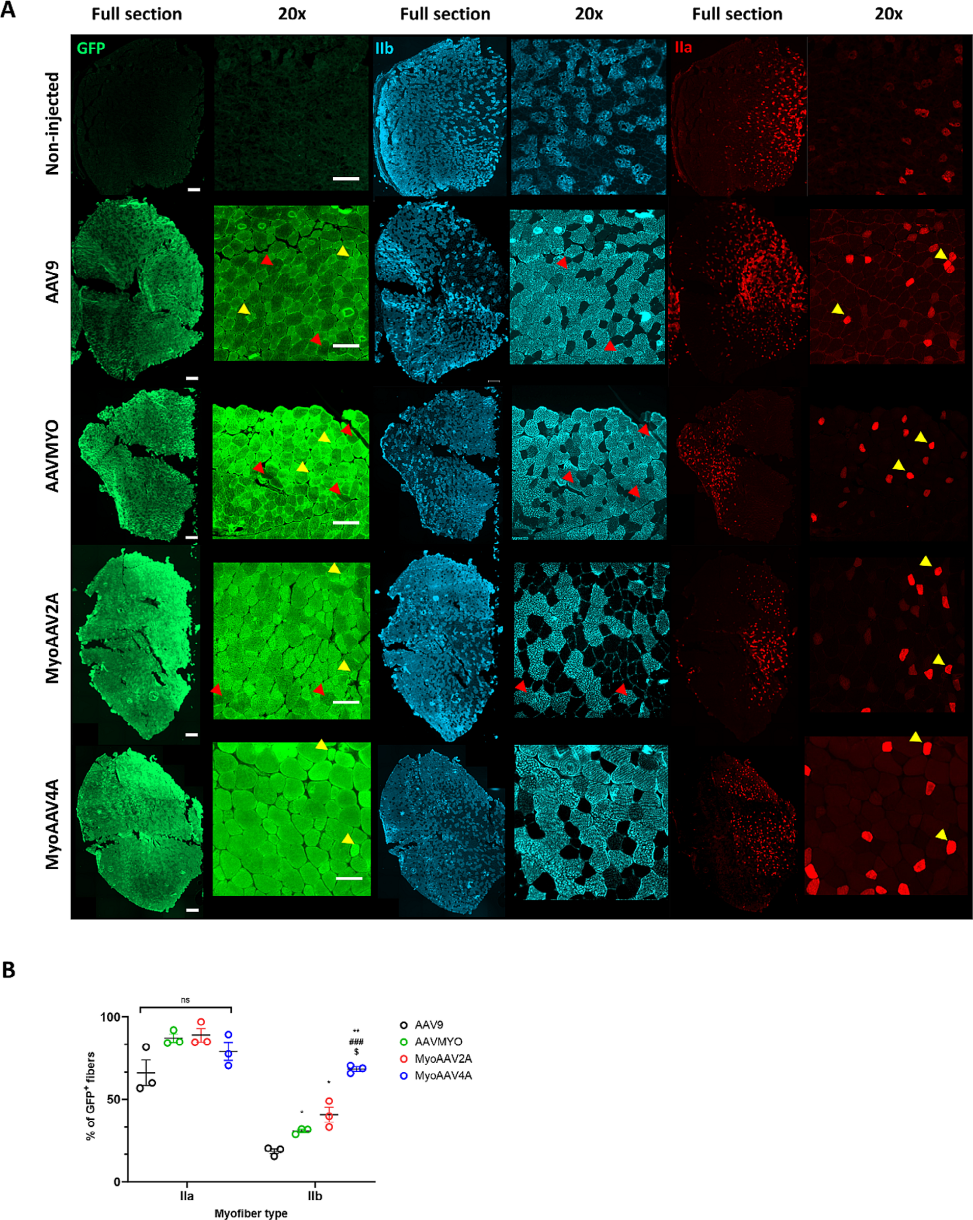
Correlation between of eGFP and myofiber IIa and IIb in AAV9, AAVMYO, MyoAAV2A, MyoAAV4A-transduced muscles. Tibialis anterior muscle sections collected 5 weeks after injection in 6-week-old C57BL/6 mice with high dose (4.5E + 13 vg/kg) of AAVMYO-, MyoAAV2A-, MyoAAV4A- and AAV9-CMV-luc-IRES-eGFP. (**A**) Fluorescent co-labeling of eGFP, myofiber IIa and IIb in TA of AAVMYO-, MyoAAV2A-, MyoAAV4A- and AAV9-injected mice. Red arrowhead and yellow arrowhead indicate respectively GFP negative IIb fibers and GFP positive IIa fibers. Scale bar is 200 μm. (**B**) Quantification of positive GFP (GFP^+^) fibers depending of the fiber type. Percentage of GFP^+^ fibers was determined by the number of fibers presenting a colocalization between GFP and IIa/IIb signal divided by the total number of myofiber IIa/IIb. Data are presented as mean values +/- SEM (*n* = 3). Two-way ANOVA with Bonferroni correction

As expected, AAV9-transduced TAs showed a higher rate of transduction in type IIa myofibers than IIb myofibers (Fig. 3A-B). All the myotropic AAVs transduced type IIa myofibers at high level (more than 80% of eGFP + fibers). Furthermore, AAVMYO, MyoAAV2A and MyoAAV4A demonstrated enhanced transduction efficiency in type IIb myofibers compared to AAV9, with the highest transduction rate achieved by MyoAAV4A under these conditions.

Thus, the better muscle transduction with MyoAAV4A in C57BL/6 mice is potentially related to their better efficiency to transduce different myofiber types.

### AAVMYO exhibits the highest transduction efficiency in 1-day post-natal injected mice

Depending on the age of onset and disease severity, patients with muscle diseases might be injected with AAV at different ages, including early stages for disease prevention. To assess if the myotropic AAVs diffuse as effectively when injected into neonates or into adult mice, both CD1 and C57BL/6 mouse strains were injected retro-orbitally at 1 day post-natal (d.p.n) with 4.1E + 12 vg/kg, and the transduction efficiency was evaluated 4 weeks later by measuring eGFP mRNA level and ex vivo luficerase activity from the TA, diaphragm muscles, heart, and liver.

**Fig. 4:**
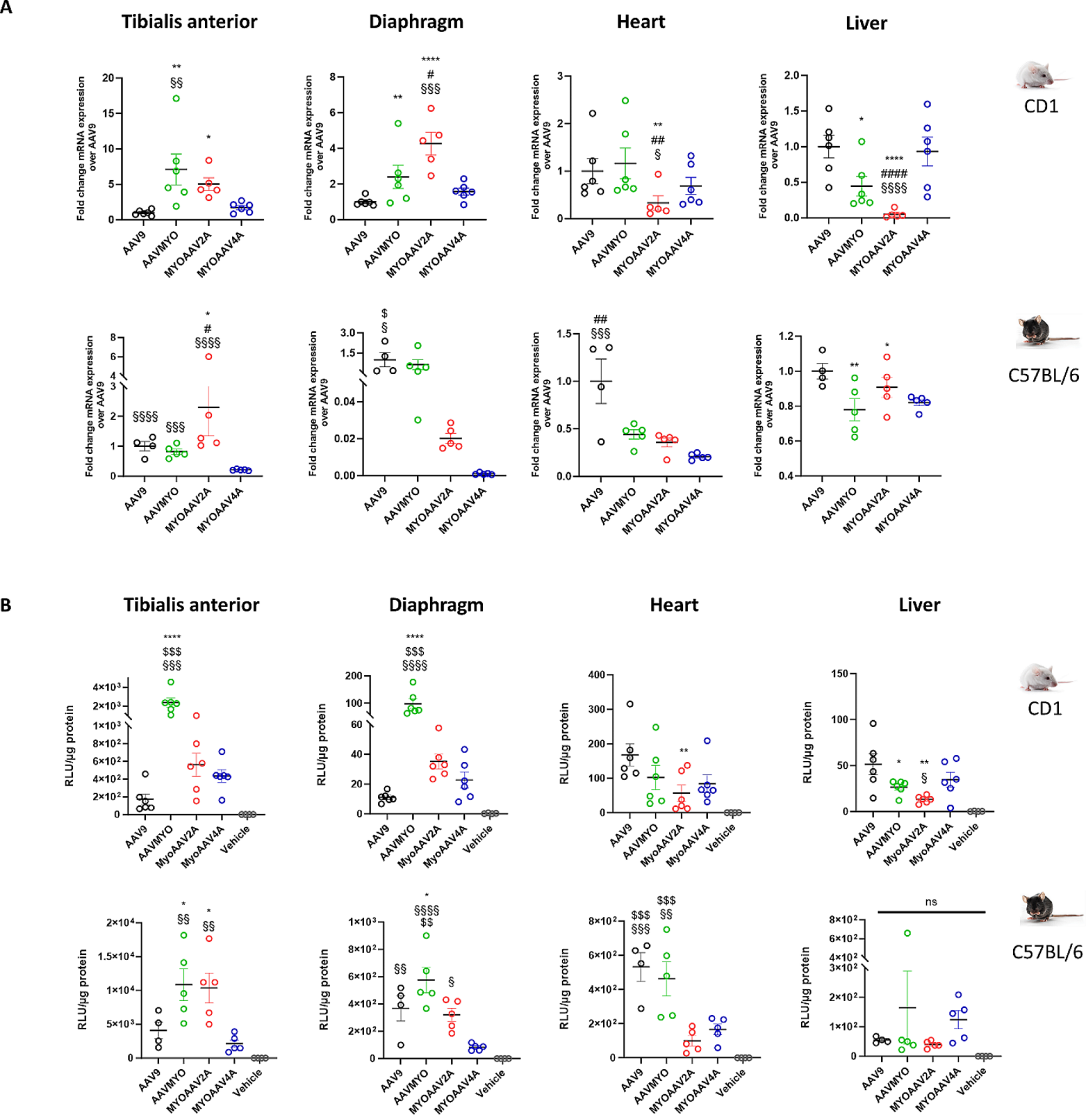
Comparison of transduction levels in different organs between AAV9 and myotropic AAVs in mice 4 weeks after retro-orbital injection at 1 day post-natal. WT CD1 or C57BL/6 mice were systemically injected at 1 d.p.n. with 4.1E + 12 vg/kg of AAV9-, AAVMYO-, MyoAAV2A-, or MyoAAV4A-CMV-luc-IRES-eGFP and analyzed 4 weeks later. (**A**) Quantification of eGFP mRNA fold change expression of AAVMYO-, MyoAAV2A-, MyoAAV4A- and AAV9-CMV-luc-IRES-eGFP in WT CD1 and C57BL/6 mice in different organs. Data are presented as mean values +/- SEM (*n* = 5–6). One-way ANOVA with Tukey correction. *p < 0.05, **p < 0.01, ****p < 0.0001 versus AAV9 ; ##*p* < 0.01, ####*p* < 0.0001 versus AAVMYO ; §*p* < 0.05, §§*p* < 0.01, §§§*p* < 0.001, §§§§*p* < 0.0001 versus MyoAAV4A. (**B**) Quantification of luciferase activity of AAVMYO-, MyoAAV2A-, MyoAAV4A- and AAV9-CMV-luc-IRES-eGFP in WT CD1 and C57BL/6 mice in different organs. Data are presented as mean values +/- SEM (*n* = 5–6). One-way ANOVA with Tukey correction. *p < 0.05, **p < 0.01, ****p < 0.0001 versus AAV9 ; $$*p* < 0.01, $$$*p* < 0.001 versus MyoAAV2A ; §§*p* < 0.01, §§§*p* < 0.001, §§§§*p* < 0.0001 versus MyoAAV4A

In CD1 mice, AAVMYO presented the highest eGFP mRNA level in TA among all AAVs, also confirmed by luciferase activity in this tissue (Fig. 4A-B). Additionally, in both mouse strains, AAVMYO presented the highest eGFP mRNA expression and luciferase activity in diaphragm (1E + 2 ± 1.7E + 1 RLU/µg protein for CD1; 5.8E + 2 ± 7.2E + 1 RLU/µg protein for C57BL/6). In C57BL/6 mice, MyoAAV2A and AAVMYO showed an increased luciferase activity in TA and diaphragm compared to AAV9 (Fig. 4B). Noteworthy, all myotropic AAVs showed a decrease of eGFP mRNA level in heart compared to AAV9. Surprisingly, MyoAAV4A presented low transduction in TA, diaphragm and heart tissues compared to all the other AAVs after injection at 1 d.p.n, unlike following adult injection (Fig. 4A-B).

In conclusion, myotropic AAVs show variations in transduction efficiency depending on the age at injection, with the highest transduction rate in 1-d.p.n-injected mice with AAVMYO in both mouse strains.

### Differential liver de-targeting for the compared AAV serotypes

The different myotropic AAVs showed increased transduction efficacy in muscles and heart. As several clinical trials with AAV8, AAV9 and AAVrh74 were halted due to liver toxicity, sometimes leading to patient death, we compared the different myotropic AAVs for liver transduction at both ages and in both strains.

In CD1 mice injected at 1 day postnatal, AAVMYO and MyoAAV2A exhibited liver de-targeting according to luciferase assay results while such de-targeting was not observed in C57BL/6 mice (Fig. 4B). However, based on eGFP mRNA levels in this mouse strain, AAVMYO and MyoAAV2A did demonstrate a de-targeting effect in the liver in both genetic backgrounds (Fig. 4A). In adult-injected mice, all serotypes tested including AAV9 displayed a lower liver transduction compared to muscles and heart (Fig. 2A-C). AAVMYO significantly de-targeted the liver in both mouse strains compared to AAV9 while this effect was observed for MyoAAV2A in C57BL/6 mice and for MyoAAV4A in CD1 mice (Fig. 2C).

Overall, the liver de-targeting effect of different myotropic serotypes was not consistently present in different mouse strains and varied depending on the age of injection, albeit AAVMYO and MyoAAV4A appeared the most promising.

## Discussion

To tackle the poor transduction efficiency and the off-target effect of AAVs used in pre-clinical and clinical developments, here we assessed different myotropic AAV serotypes that were independently created but not compared to date. Concerning leg muscle transduction, MyoAAV2A and 4 A showed the highest efficiency compared to AAV9 and AAVMYO. MyoAAV2A was also the most efficient to transduce heart. Conversely, AAVMYO was the most efficient to transduce diaphragm. In addition, we evidenced AAVMYO and MyoAAV4A presented better liver de-targeting while AAV9 and MyoAAV2A had a similar transduction for this organ. Noteworthy, these abilities highly depend on mouse strains and age at injection.

### Muscle transduction efficiency

Previous studies were conducted to assess transduction efficiency rates of natural serotypes within the mouse muscular system before the development of AAVMYO and MyoAAVs [40–43]. Here, we confirmed that all myotropic AAVs achieved a higher muscle transduction than AAV9 at the same dose in mice. We did not test AAVMYO2 and 3 due to the reported absence of enhanced muscle transduction compared to AAVMYO [44]. We report that MyoAAVs showed a better transduction efficiency in leg muscle than AAVMYO and AAV9 at a dose of 4.5E + 12 vg/kg in both mouse strains injected at 6 weeks. We also found muscle transduction varied depending on the type of muscle fiber. Indeed, Riaz et al. demonstrated that AAV9 exhibited a preference for type IIX fibers and minimal transduction in type IIb fibers [39]. Here, we found that AAVMYO, like AAV9, displayed low transduction efficiency for type IIb fibers, while MyoAAVs exhibited transduction of different myofiber types including type IIb. Noteworthy, expression of different integrins vary among myofiber types. Indeed, Weinmann et al. hypothesized AAVMYO binds α7 integrins, while MyoAAVs were suggested to prefer αV integrins [27, 28]. In heart, MyoAAV2A exhibited a higher transduction efficiency compared to AAV9, explained by it strong interaction with integrin αVβ1 and αVβ3, which is recognized to be highly expressed in both skeletal and cardiac muscles [27, 34, 45].

The fact that MyoAAVs express their transgenes in leg muscle better than AAVMYO may be based on the utilization of the DELIVER’s approach by Tabebordbar et al. which incorporated a cap gene driven by MHCK7 and CK8 promoters known for their muscle-specific activity, whereas Weinmann et al. used a ubiquitous CMV promoter in their capsid library. The utilization of a muscle-specific promoter can facilitate the identification of modified capsids that exhibit enhanced affinity for the targeted tissue.

### Important parameters for transduction efficiency

AAVs could be used either for disease prevention, as in the case of SMA, or for disease reversion, likely for myotubular myopathy. We identified several parameters that modify the transduction efficacy, most probably linked to differential diffusion or cell entry of AAVs. Variations in transduction efficiency were observed within the same mouse strain at different injection time points, and between different mouse strains at the same injection time point. While MyoAAV2A was the most proficient AAV serotype tested to transduce skeletal muscle in CD1 mice injected at 6 weeks, AAVMYO significantly outperformed all the other serotypes in CD1 mice injected at 1 day post-natal. In addition, MyoAAV4A exhibited the highest muscle transduction efficiency in C57BL/6 mice injected at 6 weeks, whereas it was the least efficient at 1-d.p.n. MyoAAV4A’s transduction efficiency could also be underestimated in our study, as the injected dose was based on the qPCR results for the determination of viral titers, which was overestimated for this serotype compared to ddPCR results (Figure S1). However, differences in transduction could be anticipated due to the fact that expression of various integrins on the muscle surface differs depending on the developmental stage. For example, α7 integrins play an important role in myoblast differentiation in the early post-natal period in mice [46, 47]. Future studies on expression levels of integrins during mouse development and comparison with different human ages should be conducted to elucidate our observed disparities and better fine-tune the best serotypes applicable to patients.

### Liver de-targeting

In addition to the best transduction efficiency for the targeted tissue, another important parameter to consider in establishing an AAV-based gene therapy is the vector’s ability to avoid transduction of the liver. Indeed, several cases of hepatic toxicity observed in clinical trials have been attributed to an excessive quantity of vector reaching the liver, or to immune system activation leading to hepatocytes’ death which represent the majority of liver cells [9]. As an organ with high blood supply, liver is especially susceptible to accumulating circulating AAVs. In this context, our study demonstrated a decrease in viral genomes, eGFP mRNA levels, and luciferase activity in 6w-injected mice with AAVMYO compared to AAV9, confirming its liver de-targeting effect. This effect was also seen with MyoAAV4A in CD1 mice and with MyoAAV2A in C57BL/6 mice albeit to a lesser extent at that age. When injected at 1 day post-natal, MyoAAV2A and AAVMYO exhibited reduced liver transduction compared to AAV9 in CD1 mice, whereas MyoAAV4A demonstrated no improvement compared to AAV9.

## Conclusion

In conclusion, this comparative analysis of various AAV9-derived vectors under the same experimental conditions at different time points in two distinct strains highlighted MyoAAV2A for best leg muscle transduction and AAVMYO for diaphragm transduction and liver de-targeting. The ability of MyoAAVs to transduce both type IIa and IIb fibers in mice indicates a promising therapeutic tool for future clinical trials. However, while all these myotropic AAVs were selected in mice, there is no guarantee that they will have similar efficiency and specificity in human. In particular, distribution and predominance of myofiber type are known to be different, with human muscles having a higher proportion of slow type fibers. Finally, this study should serve as a valuable resource for researchers pursuing to establish proof-of-concepts in preventive or curative treatments in mouse models, to ultimately lead to clinical trials for muscle disorders.

### Electronic supplementary material

Below is the link to the electronic supplementary material.

Supplementary Material 1

Supplementary Material 2

Supplementary Material 3

Supplementary Material 4

Supplementary Material 5

## Data Availability

Most of the data are included in the manuscript. Additional data supporting the findings of this study are available from the corresponding author upon request.

## Abbreviations

AAV: Adeno-associated virus
CMV: Cytomegalovirus
DMD: Duchenne muscular dystrophy
d.p.n: Day post-natal
HD: High dose
SMA: Spinal muscular atrophy
TA: Tibialis anterior
VCN: Vector copy number

## References

[1] Naso MF, Tomkowicz B, Perry WL, Strohl WR (2017). Adeno-Associated Virus (AAV) as a Vector for Gene Therapy. BioDrugs.

[2] Fang C-C (2018). AAV serotype 8-mediated liver specific GNMT expression delays progression of hepatocellular carcinoma and prevents carbon tetrachloride-induced liver damage. Sci Rep.

[3] Manno CS (2003). AAV-mediated factor IX gene transfer to skeletal muscle in patients with severe hemophilia B. Blood.

[4] Wang X, Yu C, Tzekov RT, Zhu Y, Li W (2020). The effect of human gene therapy for RPE65-associated Leber’s congenital amaurosis on visual function: a systematic review and meta-analysis. Orphanet J Rare Dis.

[5] Rajendran S et al. Targeting of breast metastases using a viral gene Vector with Tumour-selective transcription. ANTICANCER Res. (2011). 21617219

[6] Hoy SM, Delandistrogene Moxeparvovec (2023). First Approval Drugs.

[7] High-dose (2020). AAV gene therapy deaths. Nat Biotechnol.

[8] Mendell JR (2021). Current clinical applications of in vivo gene therapy with AAVs. Mol Ther.

[9] Morales L, Gambhir Y, Bennett J, Stedman HH (2020). Broader implications of Progressive Liver Dysfunction and Lethal Sepsis in two boys following systemic high-dose AAV. Mol Ther.

[10] Shieh P (2022). OP018: ASPIRO gene therapy trial in X-Linked Myotubular Myopathy (XLMTM): update on preliminary efficacy and safety findings. Genet Med.

[11] Duan D, Systemic AAV (2018). Micro-dystrophin Gene Therapy for Duchenne muscular dystrophy. Mol Ther.

[12] Chand D (2021). Hepatotoxicity following administration of onasemnogene abeparvovec (AVXS-101) for the treatment of spinal muscular atrophy. J Hepatol.

[13] Blankinship MJ (2004). Efficient transduction of skeletal muscle using vectors based on adeno-associated virus serotype 6. Mol Ther.

[14] Nam H-J (2007). Structure of Adeno-Associated Virus Serotype 8, a Gene Therapy Vector. J Virol.

[15] Gao G (2004). Clades of Adeno-Associated viruses are widely disseminated in human tissues. J Virol.

[16] Goedeker NL (2023). Evaluation of rAAVrh74 gene therapy vector seroprevalence by measurement of total binding antibodies in patients with Duchenne muscular dystrophy. Ther. Adv Neurol Disord.

[17] Tulalamba W (2020). Distinct transduction of muscle tissue in mice after systemic delivery of AAVpo1 vectors. Gene Ther.

[18] Wang D (2018). A rationally Engineered Capsid variant of AAV9 for systemic CNS-Directed and Peripheral tissue-detargeted gene delivery in neonates. Mol Ther - Methods Clin Dev.

[19] Shen S (2013). Engraftment of a galactose receptor footprint onto Adeno-associated viral Capsids improves transduction efficiency. J Biol Chem.

[20] Mao Y (2016). Single point mutation in adeno-associated viral vectors -DJ capsid leads to improvement for gene delivery in vivo. BMC Biotechnol.

[21] Bowles DE (2012). Phase 1 Gene Therapy for Duchenne muscular dystrophy using a translational optimized AAV vector. Mol Ther.

[22] Maheshri N, Koerber JT, Kaspar BK, Schaffer DV (2006). Directed evolution of adeno-associated virus yields enhanced gene delivery vectors. Nat Biotechnol.

[23] Ojala DS (2018). In vivo selection of a computationally designed SCHEMA AAV Library yields a Novel variant for infection of adult neural stem cells in the SVZ. Mol Ther.

[24] Qian R, Xiao B, Li J, Xiao X (2021). Directed Evolution of AAV Serotype 5 for increased hepatocyte transduction and retained low Humoral Seroreactivity. Mol Ther - Methods Clin Dev.

[25] Liu YB (2021). Directed evolution of AAV accounting for long-term and enhanced transduction of cardiovascular endothelial cells in vivo. Mol Ther - Methods Clin Dev.

[26] Yang L, Xiao X (2013). Creation of a cardiotropic adeno-associated virus: the story of viral directed evolution. Virol J.

[27] Tabebordbar M (2021). Directed evolution of a family of AAV capsid variants enabling potent muscle-directed gene delivery across species. Cell.

[28] Weinmann J (2020). Identification of a myotropic AAV by massively parallel in vivo evaluation of barcoded capsid variants. Nat Commun.

[29] Smith JW, Integrins DA, MECHAM RP (1994). The structural basis of integrin—ligand (RGD) Interaction.

[30] Yamada Y (2023). Structure–Activity relationships of RGD-Containing peptides in integrin αvβ5-Mediated cell adhesion. ACS Omega.

[31] Ruoslahti E, Pierschbacher MD (1987). New perspectives in Cell Adhesion: RGD and Integrins. Science.

[32] Krammer A, Craig D, Thomas WE, Schulten K, Vogel (2002). V. A structural model for force regulated integrin binding to fibronectin’s RGD-synergy site. Matrix Biol.

[33] Balasubramanian S, Kuppuswamy D, RGD-containing Peptides, Activate (2003). S6K1 through β3 integrin in adult Cardiac muscle cells. J Biol Chem.

[34] Mayer U, Integrins (2003). Redundant or important players in skeletal muscle?. J Biol Chem.

[35] Schwander M et al. ␤1 integrins regulate myoblast Fusion. Dev. Cell. 10.1016/s1534-5807(03)00118-7 12737803

[36] Sinanan ACM, Machell JRA, Wynne-Hughes GT, Hunt NP, Lewis MP (2008). αvβ3 and αvβ5 integrins and their role in muscle precursor cell adhesion. Biol Cell.

[37] Lindner L (2021). Reliable and robust droplet digital PCR (ddPCR) and RT-ddPCR protocols for mouse studies. Methods.

[38] Hämäläinen N, Pette D (1993). The histochemical profiles of fast fiber types IIB, IID, and IIA in skeletal muscles of mouse, rat, and rabbit. J Histochem Cytochem.

[39] Riaz M (2015). Differential myofiber-type transduction preference of adeno-associated virus serotypes 6 and 9. Skelet Muscle.

[40] Louboutin J-P, Wang L, Wilson JM (2005). Gene transfer into skeletal muscle using novel AAV serotypes. J Gene Med.

[41] Rivière C, Danos O, Douar AM (2006). Long-term expression and repeated administration of AAV type 1, 2 and 5 vectors in skeletal muscle of immunocompetent adult mice. Gene Ther.

[42] Zincarelli C, Soltys S, Rengo G, Rabinowitz JE (2008). Analysis of AAV serotypes 1–9 mediated gene expression and tropism in mice after systemic injection. Mol Ther.

[43] Muraine L (2020). Transduction efficiency of Adeno-Associated Virus Serotypes after local injection in mouse and human skeletal muscle. Hum Gene Ther.

[44] El Andari J (2022). Semirational bioengineering of AAV vectors with increased potency and specificity for systemic gene therapy of muscle disorders. Sci Adv.

[45] Meagher PB (2021). Cardiac Fibrosis: key role of integrins in Cardiac Homeostasis and Remodeling. Cells.

[46] McClure MJ, Ramey AN, Rashid M, Boyan BD, Schwartz Z (2019). Integrin-α7 signaling regulates connexin 43, M-cadherin, and myoblast fusion. Am J Physiol -Cell Physiol.

[47] Xiao J, Jethanandani P, Ziober BL, Kramer RH (2003). Regulation of α7 integrin expression during muscle differentiation. J Biol Chem.

