# Supplementary material for: Comparative in vivo characterization of newly discovered myotropic adeno-associated vectors
Source: Skelet Muscle. 2024 May 3;14:9. doi: 10.1186/s13395-024-00341-7 (PMC11067285; doi:10.1186/s13395-024-00341-7)
Supplement: Supplementary file 1 — Supplementary Material 1 [file 13395_2024_341_MOESM1_ESM.docx]

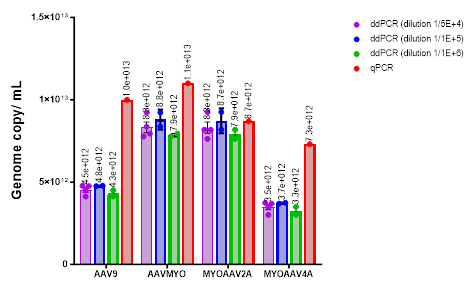


**Supplementary figure 1. Determination of viral titers of each serotype.**

Viral titers (genome copy/mL) were determined by qPCR represented in red or by ddPCR in purple (dilution 1/5E+4), blue (dilution 1/1E+5) and green (dilution 1/1E+6).
