# Supplementary material for: Comparative in vivo characterization of newly discovered myotropic adeno-associated vectors
Source: Skelet Muscle. 2024 May 3;14:9. doi: 10.1186/s13395-024-00341-7 (PMC11067285; doi:10.1186/s13395-024-00341-7)
Supplement: Supplementary file 2 — Supplementary Material 2 [file 13395_2024_341_MOESM2_ESM.docx]

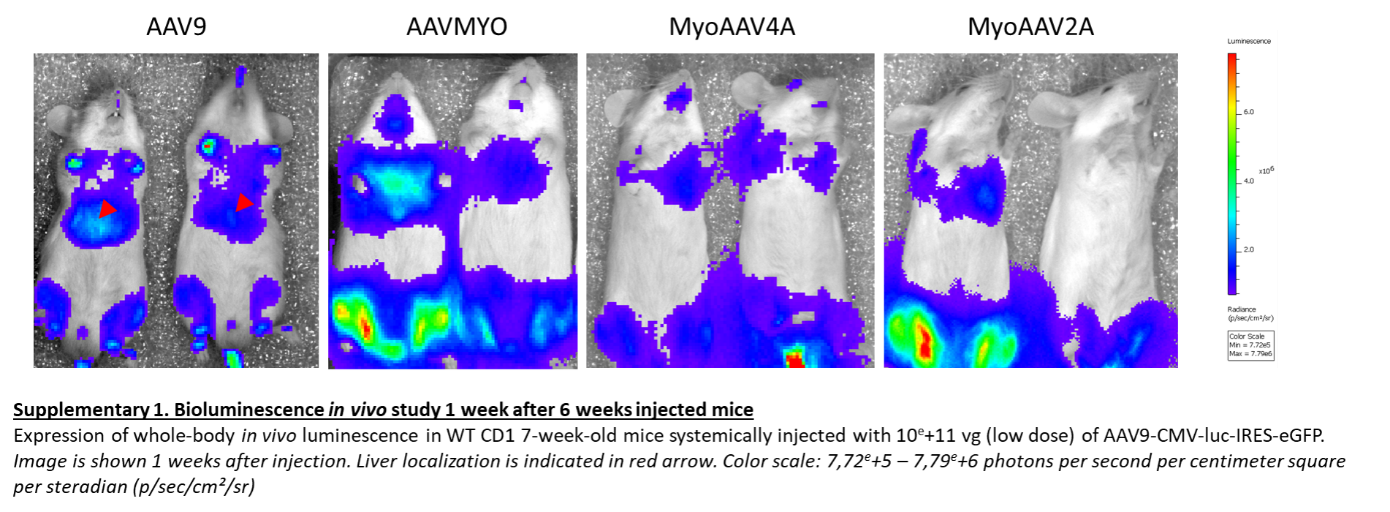


**Supplementary figure 2. In vivo bioluminescence 1 week after injection of myotropic AAVs.** Intensity of whole-body luminescence in WT CD1 7-week-old mice systemically injected at 6 weeks with 4.5E+13 vg/kg of AAV9-, AAVMYO-, MyoAAV2A-, or MyoAAV4A-CMV-luc-IRES-eGFP. Liver signals are indicated with red arrowheads. Color scale: 7,72E+5 – 7,79E+6 photons per second per centimeter square per steradian (p/sec/cm²/sr).
