# Supplementary material for: Comparative in vivo characterization of newly discovered myotropic adeno-associated vectors
Source: Skelet Muscle. 2024 May 3;14:9. doi: 10.1186/s13395-024-00341-7 (PMC11067285; doi:10.1186/s13395-024-00341-7)
Supplement: Supplementary file 3 — Supplementary Material 3 [file 13395_2024_341_MOESM3_ESM.docx]

**Supplementary figure 3. Comparison of *in vivo* luminescence levels between male and female in AAV9 and myotropic AAVs injected adult mice.**

Quantification of *in vivo* luminescence in mice injected via tail vein with AAV9-, AAVMYO-, MyoAAV2A-, MyoAAV4A- at 4.5E+12 vg/kg taken at 11 weeks. Luminescence is quantified by measuring the average radiance (p/sec/cm²/sr). Data are presented as mean values +/- SEM (n= 4-13). Two-way ANOVA with Bonferroni correction.
