# Supplementary material for: Comparative in vivo characterization of newly discovered myotropic adeno-associated vectors
Source: Skelet Muscle. 2024 May 3;14:9. doi: 10.1186/s13395-024-00341-7 (PMC11067285; doi:10.1186/s13395-024-00341-7)
Supplement: Supplementary file 4 — Supplementary Material 4 [file 13395_2024_341_MOESM4_ESM.docx]

**Supplementary figure 4. Comparison of *in vivo* luminescence levels between two different routes of injection of AAV9 and myotropic AAVs in 5w old mice.**

Quantification of in vivo luminescence in mice injected with AAV9-, AAVMYO-, MyoAAV2A-, MyoAAV4A- at 4.5E+12 vg/kg taken at 11 weeks. Luminescence is quantified by measuring the average radiance (p/sec/cm²/sr). Data are presented as mean values +/- SEM (n= 4-13). Two-way ANOVA with Bonferroni correction.
