# Supplementary material for: Comparative in vivo characterization of newly discovered myotropic adeno-associated vectors
Source: Skelet Muscle. 2024 May 3;14:9. doi: 10.1186/s13395-024-00341-7 (PMC11067285; doi:10.1186/s13395-024-00341-7)
Supplement: Supplementary file 5 — Supplementary Material 5 [file 13395_2024_341_MOESM5_ESM.docx]

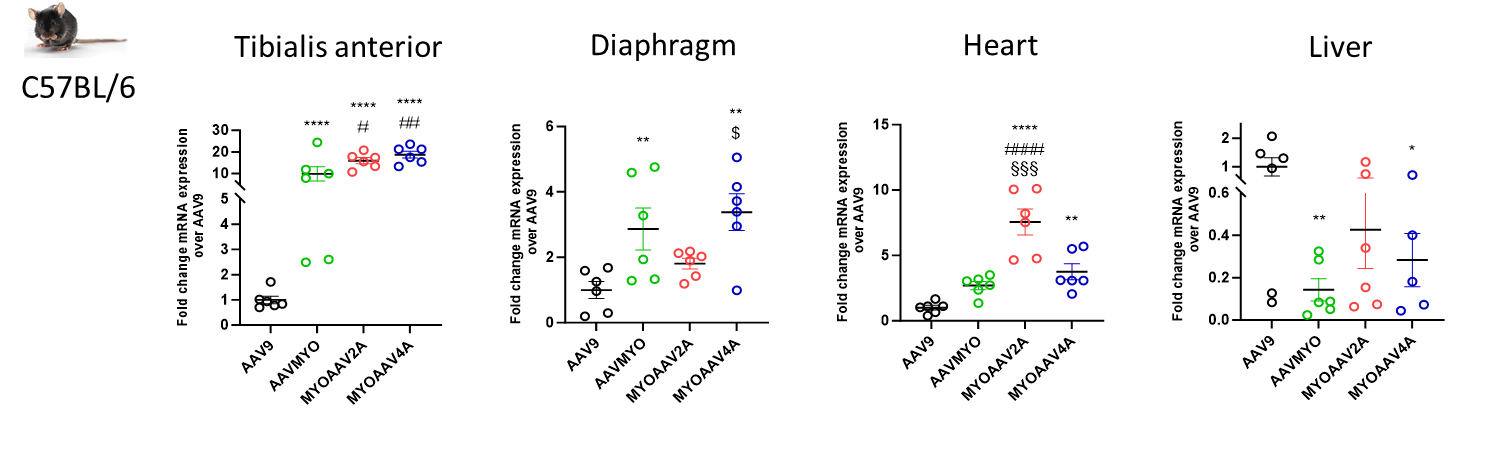


**Supplementary figure 5. Ex vivo comparison of transduction efficiency between AAV9 and myotropic AAVs at high dose in C57BL/6 mice.** Quantification of eGFP mRNA fold change expression of AAVMYO-, MyoAAV2A-, MyoAAV4A- and AAV9-CMV-luc-IRES-eGFP in WT C57BL/6 mice injected at high dose (4.5E+13 vg/kg) in different organs. Data are presented as mean values +/- SEM (n= 6). One-way ANOVA with Tukey correction. *p<0.05, **p<0.01, ****p<0.0001 versus AAV9 ; #p<0.05, ##p<0.01, ####p<0.0001 versus AAVMYO ; $p<0.05 versus MyoAAV2A ;§§§p<0.001 versus MyoAAV4A.
